# Building a synthesis-ready research ecosystem: fostering collaboration and open science to accelerate biomedical translation

**DOI:** 10.1186/s12874-025-02524-2

**Published:** 2025-03-10

**Authors:** Alexandra Bannach-Brown, Torsten Rackoll, Malcolm R. Macleod, Sarah K. McCann

**Affiliations:** 1 QUEST Center for Responsible Research, Berlin Institute of Health at Charité – Universitätsmedizin Berlin, Charitéplatz 1, 10117 Berlin, Germany; 2 Centre for Clinical Brain Sciences, The University of Edinburgh Medical School, Edinburgh, UK

**Keywords:** Evidence synthesis, Open science, Systematic review, Meta-analysis, Preclinical research, Animal models

## Abstract

In this review article, we provide a comprehensive overview of current practices and challenges associated with research synthesis in preclinical biomedical research. We identify critical barriers and roadblocks that impede effective identification, utilisation, and integration of research findings to inform decision making in research translation. We examine practices at each stage of the research lifecycle, including study design, conduct, and publishing, that can be optimised to facilitate the conduct of timely, accurate, and comprehensive evidence synthesis. These practices are anchored in open science and engaging with the broader research community to ensure evidence is accessible and useful to all stakeholders. We underscore the need for collective action from researchers, synthesis specialists, institutions, publishers and journals, funders, infrastructure providers, and policymakers, who all play a key role in fostering an open, robust and synthesis-ready research environment, for an accelerated trajectory towards integrated biomedical research and translation.

## Why we need synthesisable research

Individual research articles contribute to the accumulation of scientific knowledge. However, single pieces of evidence are rarely sufficient for effective decision-making in biomedicine. Evidence synthesis is required to integrate the vast, and at times contradictory, biomedical literature. Systematic review is an evidence synthesis methodology involving the systematic identification, appraisal, and integration of all studies addressing a specific research question, crystallising our understanding of a topic. It facilitates the detection of false leads and new relationships, yielding more precise and reliable evidence than individual studies. Systematic reviews are used routinely in clinical medicine to inform healthcare practice, guidelines, and policy. Increasingly, systematic reviews are used in preclinical biomedicine, including in vivo and in vitro research, to identify more clearly what is currently known, how reliable the evidence is, and where future research is needed [1]. They can help guide decisions aligned with the 3Rs (Replacement, Reduction, Refinement), regarding when or how new animal experiments should be conducted, or when preclinical testing should advance to the clinic. From the researcher’s perspective, the inclusion of their data in a systematic review clearly demonstrates a pathway to impact for their work.

Despite these benefits, barriers at multiple stages of the research process currently inhibit the effective conduct and utilisation of evidence syntheses. In this article, we highlight some of these barriers and propose solutions for how biomedical research can become “synthesis-ready”, through embracing open scholarship and involving stakeholders from researchers to publishers to funding bodies. While many of the barriers we discuss are shared with clinical and other research types, there are issues peculiar to, or exaggerated in, preclinical research due to differing experimental structures and reporting practices. These include a vast existing literature, an increasing rate of scientific publication, and the dispersion of related research across manifold individual journals and databases [2]. These features emphasise the pressing need for effective evidence synthesis within preclinical biomedicine.

## The current research system and barriers to evidence synthesis

Primary preclinical evidence is commonly generated by individual groups of laboratory-based researchers, whereafter a separate group of evidence synthesists attempts to locate and integrate these findings in a systematic review or other type of synthesis. The disjointed nature of current practices results in large inefficiencies, and numerous barriers can hinder the structured processes of a systematic review. It can take many years before reliable evidence and consensus on a topic are established [3].

Systematic review aims to synthesise all available evidence on a specific research question. In preclinical research this usually includes a large number of small studies describing few animals or samples. This contrasts with systematic reviews of clinical research, which generally involve a relatively small number of clinical trials, each with many participants. The structured steps of a systematic review involve (i) preparing a protocol of the proposed methodology, (ii) a systematic search across multiple bibliographic databases to identify all potentially relevant literature, (iii) removal of duplicate citations to establish a library of unique publications, (iv) screening citations for relevance to the research question, based on pre-specified inclusion and exclusion criteria, (v) extracting key study design characteristics and quantitative outcome data from included studies, (vi) assessing the quality of included studies and their risks of bias, (vii) synthesising all relevant data, descriptively or quantitatively in the form of meta-analysis, and (viii) transparent publication of the findings. For a full description of the systematic review process, please see comprehensive guides published elsewhere [4–6].

In order to most effectively perform systematic reviews, synthesists are increasingly employing automation tools, where high quality systems are available, to support the human process of gathering, processing, and synthesising evidence. Multiple steps in the systematic review process can now be supported by automation tools, though not all [7]. In particular, several tools exist to assist researchers identify relevant literature, and tools to extract key information and assess study quality are under development [8]. To apply automated tools, data must be openly accessible and machine-readable, structured in a format that is readily processed by a computer without human intervention.

Below, we describe specific systematic review processes where barriers to evidence synthesis are most evident. In many instances, these barriers also affect the ability to effectively automate review processes, increasing the human burden and decreasing the efficiency with which systematic reviews can be conducted.

## Systematic searching and identifying unique information

The foundation of a comprehensive systematic review is identifying and accessing information from relevant research artefacts. At present, most take the form of scientific papers, however a research artefact can also refer to an experimental protocol or a dataset resulting from an experiment. Authors publish papers in journals that may be more or less specific to their field of research, meaning that papers on the same topic can be dispersed across a wide range of journals. Over 8500 biomedical journals are indexed in multiple, often overlapping, bibliographic databases e.g., PubMed [9], Embase [10], and Web of Science [11], making it difficult to know exactly where or how broadly to search for research on a given topic.

Systematically searching for biomedical literature involves generating lists of terms relevant to the research question that may be located in an article’s title, abstract, or structured metadata. However, key information about the experimental design (e.g., the paradigms used in the experiments, interventions tested, test subjects used) are often not fully reported by authors, as abstracts are restricted summaries and not all outcomes or experiments are necessarily described [12]. Some information may be available as structured metadata, including dictionary terms like Medical Subject Headings (MeSH), the controlled vocabulary used for indexing articles in PubMed, however these vocabularies are not standardised across bibliographic databases. This has implications for systematic searches, where evidence may be missed if an intervention or outcome assessment is not described within these limited metadata fields. Currently, this means that broad, low-specificity searches are often needed to capture all potentially relevant information, resulting in a high number of irrelevant search returns that must be screened.

The quality or comprehensiveness of a search can vary because institutions and individual researchers do not have access to the same databases and therefore to the same range of evidence. A further challenge is that, on occasion, authors describe the same research, or substantially overlapping research, in more than one paper. These duplicate publications may be related to the process of splitting data derived from a single study into multiple smaller publishable units (commonly referred to as “salami-slicing”). Without sound justification and transparency, these practices make it difficult to understand where research is referring to the same or a different experiment, and where data are describing endpoints from the same animals. Including duplicated positive data in a meta-analysis, for example, can result in exaggeration of estimated treatment efficacy.

## Removing duplicate citations

When searching across multiple databases, duplicate records that describe the same paper are usually found. Several options for automated detection and removal of duplicate records exist [13]. However, it can be difficult for these tools to ascertain whether two records are referring to the same piece of work because the quality and format of publication metadata available from different bibliographic databases varies greatly. At the time a search is carried out, one bibliographic database may hold, for example, an electronic publication ahead of print version (ePub), while another may hold the “printed” version, sometimes with a different year of publication. This makes the process of automated identification of unique records and removing all duplicate records difficult. For limited search returns with a small number of potential duplicates, manual deduplication is not troublesome, but this becomes less feasible as the number of search results, and possible duplicates, increases into the thousands or tens of thousands.

## Linking relevant information

Systematic review authors usually want to identify the most up-to-date evidence, and are increasingly searching for the latest literature in preprint servers, a practice accelerated by the COVID-19 pandemic [14]. A large proportion of preprints are subsequently published in peer-reviewed journals [15], resulting in different public versions of a single study. Preprints can be linked with the final publication using relationship metadata (e.g., through CrossRef (RRID: SCR_003217)), or title- or author-based matching. However, titles sometimes change and there may be a time delay to this formal link and the resulting update to record metadata across databases. When several versions of a publication exist (e.g., preprint, author’s own copy, and ePub ahead of print), it can be difficult to establish whether these versions are identical or if, for instance, experiments have been added (or removed) in the transition through peer review to published paper [16]. Tracking changes is important to assess study quality; to illustrate, if an additional experiment was conducted or experimental details removed based on peer review feedback, this can have implications for selective outcome reporting (described further below).

## Accessing full texts and extracting relevant data

To read the detailed information described within a research paper, one needs access to the full-text. Not all research papers are published under open access, and the full-text version may be behind a paywall. Again, institutional or individual access to databases of full-text records varies, and therefore not all researchers have access to the same range of evidence.

Full-text articles are presented in varying layouts, most frequently as PDFs but sometimes as structured HTML or XML, formats used for displaying information in a web browser (e.g., indexed articles in PubMed Central). PDFs are not standardised across journals and machine-readability varies. Papers published in online formats are also not standardised and so, while machine-readable, automating processes such as extracting study design characteristics or identifying linked supplementary data is not straightforward.

In the vast majority of published articles, quantitative data for individual samples, animals or subjects are not reported. Instead, the summary data are presented in graphs (often with an inappropriate graph type for the data [17]) in a static image format in the PDF or web page. Quantitative data are vital to evidence synthesis, both to verify the claims made in an article, and to pool and summarise using meta-analysis. To extract these data from figures presented as static images, graphs can be measured using desktop rulers (e.g [18]). This is usually a manual and incredibly time-consuming process that can be imprecise and introduce human error [19].

## Accessing raw data

The availability of raw data (e.g., the files used for data analysis) eliminates the need to manually extract summary level data from graphs or tables. Additionally, access to raw individual subject or animal level data allows more detailed verification and more sophisticated evidence synthesis techniques. However, raw data are rarely published. More recently, funders and journals are implementing recommendations or mandates for making experimental protocols and raw data supporting an article openly available (e.g., eLIFE and F1000 instructions to authors for Digital Object Identifiers (DOIs) for datasets), but this is not yet standard procedure. If data are provided, often as an appendix or in a separate data repository, the links to these datasets or protocols are not cited in a standardised way and can be found variously e.g., in the body of the text of the publication, in a data availability section, or in the references. This makes it difficult to locate and access supporting documents systematically [20], which impacts the efficiency of meta-analysis and re-analyses of findings [21].

Although the statement “*data available upon request”* is now commonly regarded as not fulfilling the criteria for open data, external links to datasets or protocols may break over time [20, 22]. Data deposited in a separate repository are not always accessible [20]. Where data are accessible, the data format may not allow for readability without proprietary software, and data are often not presented in a standardised or FAIR-compliant way. FAIR refers to the Findability, Accessibility, Interoperability, and Reusability of digital assets [23]. In particular, datasets often lack metadata and a data dictionary describing the variables present in enough detail to enable reuse by persons not intimately familiar with the experiment [24].

## Assessing study quality and biases

Currently, papers describing primary experiments often lack a pre-registered study protocol or transparent reporting of an experiment, so a reader cannot be certain that all measured outcomes have been reported. Not doing so can lead to selective outcome reporting bias [25–29]. This can occur when a set of outcomes, e.g., a battery of neurobehavioural tests, are assessed but only a subset of (often positive) results are reported, or non-significant outcomes are reported with *“data not shown”*, preventing their inclusion in syntheses. Publication bias, which occurs when an entire study or dataset – usually with null or negative findings – remains unpublished, is a recognised problem in preclinical systematic reviews and can lead to effect overestimates in meta-analyses [27]. However, this bias is also difficult to measure accurately in the absence of pre-registered study protocols.

Incomplete reporting of methods and data has a profound impact on the veracity and comprehensiveness of evidence syntheses and results in an inability to accurately assess study designs for quality and risks of bias. Very often, a preclinical risk of bias assessment will result in an “unclear” determination of risk, providing scant ability to assess the level of confidence in synthesised data and resulting conclusions [30].

Together, these factors contribute to a situation where preclinical systematic review research takes so long that many reviews are out of date when published. They may be inconclusive or contain biases due to widespread suboptimal reporting, limiting their potential to accurately inform collective scientific understanding and succeeding steps in the research pipeline.

## What do we need to be synthesis-ready in preclinical research?

Practices at each stage of the research process can be optimised to enable the conduct of timely, accurate, and comprehensive evidence synthesis. This includes open science practices such as registering planned experiments in protocols and registries, reporting detailed experimental methods, and the open dissemination of data in a format amenable to synthesis. Concretely, a number of factors could be improved concurrently to bring about change, and each stakeholder in the academic ecosystem can play a valuable role (Table 1).

## Supporting synthesis-ready research and reporting practices

### Study design protocols and data management

Study design protocols detail the proposed design and methodology for an experiment. Publicly pre-specifying these protocols enhances scientific transparency and rigour and protects the researcher against concerns regarding selective reporting of results and other questionable research practices. It allows systematic reviewers to evaluate potential biases by checking concordance between the methods as planned and as executed, to assess whether discrepancies are addressed adequately. While registries for animal study protocols exist, uptake of this practice has been slow [31, 32].

Journals and publishers can also support the publishing of protocols and planned methods as separate research artefacts and support the “Registered Reports” publishing format to ensure that the results of high-quality experiments are published, regardless of their p-value or assumed impact, and to incentivise methodological rigour. An open question remains: which player in the research ecosystem should be responsible for reviewing study design protocols as separate research artefacts? Some propose this role should be extended to Institutional Animal Care and Use Committees (IACUC) [28]. Another option may be that peer review is formalised in the journal system, as is being realised with platforms such as PLOS and Nature Protocols [33, 34]. While not a replacement for peer review, automated tools can be integrated into editorial software to improve usability for reviewers and editorial staff [35, 36].

There is also an integral role for funders to nurture good quality, useful science. Funders can request best practices at the very beginning of the research process, the grant application. Requiring and reviewing detailed protocols, data management plans, and data curation plans at the grant application stage ensures researchers have considered these aspects prior to the start of experiments. Funders can ensure there is appropriate funding allocated to maintain good data management practices throughout the lifecycle of a project. Support must also be provided to train researchers in open science and data management practices. Here, institutions can play a role in ensuring students and scientists are appropriately trained in aspects not traditionally included in curricula, such as accessibility, metadata, machine-readability, and preclinical evidence synthesis.

### Reporting guidelines

The quality of reporting in research articles is key to understanding how a study was conducted, and to enable evidence synthesis and the exploration of between-study variability (heterogeneity). Reporting guidelines for preclinical research are widely available, e.g., the ARRIVE 2.0 guidelines for animal research [37] and the MDAR (Materials Design Analysis Reporting) Framework [38]. Initiatives like RRIDs (Research Resource Identifiers; [39]) can further assist researchers to accurately and transparently document and report materials that were used during experiments (e.g., cell lines, antibodies, software, and tools). More recent advances towards open methods and improved reproducibility include recommendations for reusable step-by-step protocols, or standard operating procedures [40]. These protocols contain a very detailed sequence of operations that allow others to reproduce a method. Where materials and methods are consistently described in detail, study design and quality elements that contribute to heterogeneity in observed effects can be identified when conducting meta-analyses.

Journals and editorial processes play a key role in mandating adherence to best reporting practices, including supporting authors during submission, and supporting reviewers and editorial staff during the peer review process. Many journals have endorsed reporting guidelines, but research has shown that additional measures are required during the editorial process to improve reporting quality in publications [41]. Further journal-supported research is necessary to evaluate strategies to achieve greater improvement in reporting practices. Simply endorsing adherence to certain checklists in author submission guidelines does not ensure compliance and is no longer sufficient [42].

Further improvements can be made in reporting and disseminating completed experiments, even when results are non-significant. Guidance on how to publish negative and non-significant results is available from the file drawer data liberation effort (fiddle) tool (RRID: SCR_017327 [43]).

## Establishing research infrastructure and frameworks

### Data repositories

Data need to be reported for all outcomes and for all experiments to allow an unbiased assessment of the literature. The next step in open data for synthesis-ready research is the use of centralised data storage locations (i.e., data repositories) and adherence to common metadata standards for reporting experimental data. The benefits of open data for scientific trust and accountability have been recognised [44, 45]. There has been an increase in the availability of all-purpose data storage platforms (e.g., Open Science Framework and Zenodo); however, data deposition without adequate curation limits the benefits to data reuse and improving reproducibility [46].

Several research domains have taken the lead in their respective communities and formed working groups of multiple stakeholders to define minimum metadata standards e.g., Research Data Alliance [47], the International Neuroinformatics Coordinating Facility [48], and Open Data Commons for Spinal Cord Injury (ODC-SCI [46]). Repositories such as ODC-SCI allow the upload of animal and human experimental data in a flexible manner, with balance between human and machine readability [46]. Shared data are FAIR-compliant and receive a DOI when automated error checks have been completed and the dataset is made public. These approaches have been developed and coordinated with widespread community engagement, with the aim of facilitating culture change.

### Metadata

Curation that supports FAIR data requires structured metadata describing the conditions under which those data were collected, including materials used in the experiment. Structured metadata about the context and quality of the research data that researchers use allows computer programmes and scripts to conduct automated sorting and prioritising tasks (principle F2 of FAIR [23]). This enables key steps in any evidence synthesis process, including grouping research describing similar diseases, interventions, mechanisms, or outcomes [25]. Minimum metadata standards allow for repurposing data from previous experiments, to support the 3Rs principles of animal use in biomedical research [24].

Initiatives to advance data sharing and data repositories need to be accompanied by resources and education about data curation to ensure the full power of data is unlocked. Setting domain-wide data standards is no small feat. There is substantial cross-discipline work to be done to build common data elements (CDEs) and ontologies, approaches to naming and defining variables and their relationship to one another; to ensure that minimum data formats and metadata standards meet the needs of research teams across biomedical sciences (e.g., cancer, immunology, stroke); and to guarantee that there is interoperability in multidisciplinary domains.

## Promoting synthesis-enabling publishing and indexing practices

### Machine readability

Research papers, datasets, records, and other artefacts must be openly available and machine-readable to enable the harnessing of automation tools in evidence synthesis. Currently, custom automation tools can be applied to individual steps of the synthesis process or to individual research artefacts, e.g., querying a bibliographic database, or tools to convert PDF articles to machine readable formats. However, scholarly articles are primarily published as unstructured data and not all research artefacts are machine-readable. Even for those that are e.g., HTML or XML, there can be challenges in conversion to a standard useable format that gives ready access to text and to data presented in tables or figures. Current analysis tools therefore cannot be readily applied on all research papers and considerable manual human effort is still required to synthesise a body of evidence.

There are efforts from the research community to expand the use of standardised formats that allow the interoperable exchange of scholarly articles [49]. Further, frameworks for web-first paper formats in XML, JSON, and HTML languages are also in advanced development [50]. Optimisation and integration of these approaches will require close cooperation between research infrastructure providers and publishers.

### Data availability and veracity

Requiring that raw data accompany a research paper and publishing it open access in a machine-readable format allows the automatic production of human-readable visualisations (e.g., graphs), as well as making the underlying data available for secondary processing. Some journals already support this format, where executable analysis code for rendering graphs and the underlying data are made available so that figures can be created reactively and reproducibly in the browser [51, 52]. Widespread availability of this format would allow the seamless integration of relevant raw data into evidence syntheses, increasing substantially their efficiency. The availability of individual subject or animal data enables meta-analysis at the level of individuals, rather than studies, a more sophisticated technique that facilitates a richer understanding of variability [53]. Data availability is not without ethical and legal considerations, however operating under the “as open as possible, as closed as necessary” principle with transparent justification ensures catering to most eventualities [54].

The quality and interoperability of raw data determines their value for reuse. Review or quality assurance to ensure accuracy and usability of datasets and analysis code is also an open topic. The burden cannot fall solely on peer reviewers, as it is time-consuming to appropriately assess datasets and code and can be difficult without appropriate training. Strategies including computational reproducibility review at institution and publisher level are currently being investigated and evaluated for effectiveness and feasibility [55, 56].

Curated repositories such as ODC-SCI, which support researchers to ensure data are FAIR-compliant and useful to the community beyond the planned primary experiments, reduce the burden on downstream processes to assess reusability. However, for this and other data management activities, appropriate training and funding must be provided, as these can be time-consuming to execute well.

### Linking research artefacts

A consistent mechanism is needed to link all research artefacts related to a single study with one another: pre-registered protocols, detailed experimental procedures for all paradigms and assays, raw data deposited from all experiments, data analysis scripts, the curated “human-readable” publication, and corresponding peer review comments. Linking data and results with fully described protocols enables a complete understanding of how data are collected, ensuring data veracity and value. Linking via quality metadata standards, to ensure correct versioning and appropriate connections between artefacts [57], allows for automatic querying and data retrieval, and addresses the issue of having multiple DOIs for the same study (e.g., for manuscript (versions), datasets, and code).

A first step to ensure associated artefacts are linked in a standardised way, is to build on existing database and search engine metadata fields, e.g., PubMed “Secondary Source ID” or Web of Science “Associated Data” fields [20]. Publishers and journals should play a key role in ensuring that all related research artefacts are linked, also at a metadata level, and ensuring article metadata is up to date in bibliographic databases. DOIs or web links to a dataset or other relevant artefact that are placed inside article text by authors are often left unstructured by the publisher and provide no linking mechanism at the metadata level. The widespread lack of structured and standardised data in scholarly publishing highlights a large information gap that is preventing us from reaping the benefits of modern data formatting practices.

New models of publishing are being tested to link research artefacts pertaining to the same experimental study. These include threaded publications, which enable the curation of outputs from individual steps of the research process e.g., Octopus (www.octopus.ac) and ResearchEquals (www.researchequals.com). Further, journals such as eLife can utilise their existing model of ‘publish, review, curate’ to collate not only collections of themed research articles (Technology at eLife, 2022), but collections of documents pertaining to the same experimental study. These models and tools ensure that all published research artefacts are linked, and information is traceable. Strong linking can support tracking of pre-registered animal studies from protocol registries (e.g., preclinicaltrials.eu and animalstudyregistry.org). Ensuring registrations are kept up to date will allow for “evidence surveillance” to diagnose false leads early, identifying which studies have been terminated and where unpublished negative and neutral data are stored. Together, pre-registration and curated data repositories can help tackle publication bias in preclinical systematic reviews [58, 59].

### Supporting automation

Being “synthesis-ready” in preclinical biomedical research means having the ability to effectively leverage automated approaches such as text-mining, machine learning, and artificial intelligence (AI). Automated processing of printed text can be conducted using various approaches, which have evolved with increasing computational power, from basic text-mining to Natural Language Processing (NLP), lately used to train large language models (LLMs). Generative AI tools like ChatGPT have recently captured public imagination and are also under investigation to support systematic review [60].

Improved metadata standards will support further advances in evidence synthesis by allowing for the use of neural networks and other unsupervised machine learning to identify new patterns in biomedical data and potentially unlock novel biological insights. These emerging techniques are currently being used in e.g., genomics and physics to identify new scientific leads [61].

While promising, there are currently hurdles to the development and utilisation of these tools, in addition to the aforementioned factors related to machine-readability, accessibility, and interoperability. Automatically processing and mining data from the scientific literature for e.g., creating LLMs, means being able to accurately extract facts from varied presentation of findings (text, images, and data) in combination, often from complex studies involving multiple experiments in single scientific outputs. Validation of automated output for specific use cases is also necessary. Especially when utilising automation tools for evidence syntheses for e.g., regulatory decisions and key healthcare policy making, there is a need to ensure tools are performing to the level of gold standard; that of two expert human reviewers. Especially in preclinical research, training data can be of suboptimal quality and, currently, the additional workload of validating the performance of tools can be prohibitive. When utilising tools for slightly different use-cases, researchers need to further validate performance levels [62]. Where tools are developed under specific conditions, limited resources within a systematic review team may exist to (i) access tools, (ii) implement tools that require programming expertise, and (iii) validate tools for their specific use-case.

**Table 1:** Stakeholders and suggested actions to facilitate synthesis in preclinical research

Stakeholder	Action
Researchers	• Pre-register study protocols • Publish step-by-step methods protocols • Follow appropriate reporting guidelines • Publish all results (including neutral and negative results) • Make data and analysis code openly available in machine-readable formats, using field-specific repositories, where available • Utilise preprint servers for making research available in a timely manner • Use community-endorsed documentation and metadata standards including common data elements (CDEs) and ontologies
Evidence synthesists	• Engage with stakeholders to improve how evidence summaries are curated and visualised for different audiences • Develop and integrate automated tools to streamline and improve the accessibility of evidence synthesis research
Institutional support	• Provide open science and research data management training • Support synthesis-related infrastructures e.g., as core facilities
Publishers, journals & editorial	• Support protocol publishing formats e.g., Registered Reports • Mandate adherence to best reporting practices • Automated reporting quality checks to support peer-review • Require raw data deposition and perform data veracity and analysis script checks • Support publication formats and practices that allow linking of related research artefacts • Participate in research to improve reporting quality and data sharing
Funders	• Require a systematic summary of the field with appropriate evidence synthesis techniques, prior to funding new research • Call for protocols and data management plans at grant submission and provide appropriate evaluation • Provide appropriate funding to maintain good data management and sharing practices throughout the lifecycle of a project • Implications for non-compliance with transparent reporting and data sharing • Sustainable funding of research and synthesis infrastructure e.g., databases
Database & infrastructure providers	• Adequate linkage formats for protocols, data, analysis code, preprints and publications • Endorse and facilitate the use of domain-specific or domain-agnostic metadata standards for non-experts
Policymakers & regulators	• Build a legislative framework for EU based research to facilitate faster implementation of the proposed elements • Legal responsibility for institutions and researchers for not depositing and sharing all data gathered during research cycles • Mandating open data for every publicly funded animal experiment
Coordinated efforts	• Support for open science practices at every stage of the research lifecycle • Platforms to enable open discussions across multiple stakeholders • Community-led decisions on data ontologies and standards • A culture of data sharing, reuse, and synthesis

## Connecting the ecosystem, future directions and supporting technologies

Engaging stakeholders to facilitate changes in practice and culture, combined with key developments in research and reporting, infrastructure, and publishing are vital to unlock data and facilitate the trajectory towards a synthesis-ready ecosystem.

**Fig. 1:**
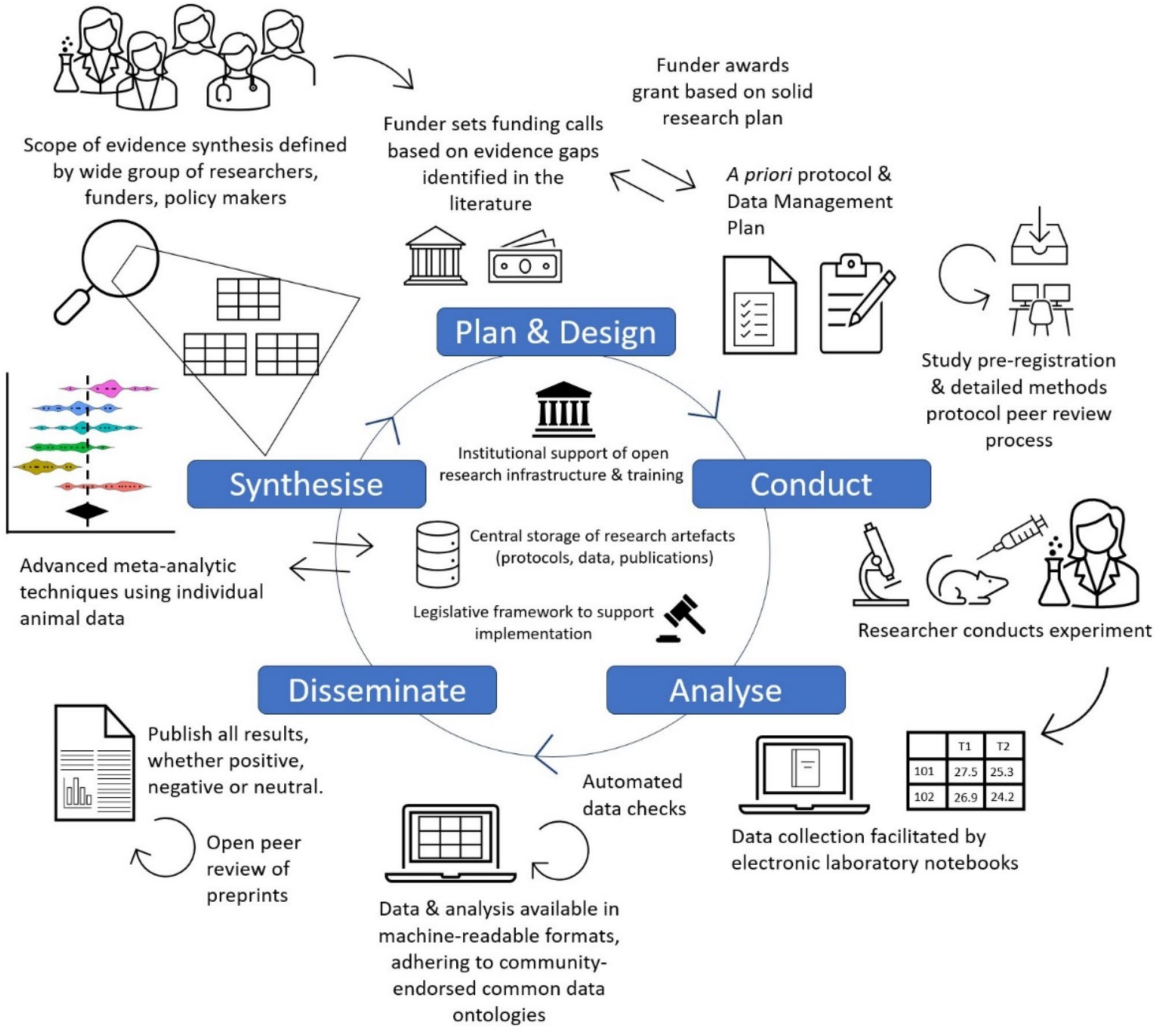
A synthesis-ready research ecosystem where processes throughout the research lifecycle, from designing, conducting, and analysing to disseminating and synthesising research, are open and robust. The roles of key stakeholders in the system are highlighted, including funders, researchers, research institutions, publishers, infrastructure providers, and policymakers

A synthesis-enabled future can be envisaged where research artefacts critical to evaluating a study, including study and methods protocols, raw data, and code are published in machine-readable, open-access formats. These are linked with one another and any related paper(s), along with minimum meta-data congruent with domain ontologies. Automated checks for machine-readability and data veracity are carried out. Researchers use community-developed CDEs and related methods, and outcomes are easily identified within and across fields. Studies or data relevant to a particular topic can be automatically detected and tagged with specific study design and quality attributes. Data from similar experiments are easily synthesised to inform pressing research questions important for decision-making and translation in biomedicine (Fig. 1).

This filtering and evaluating of relevant literature can increase the efficiency with which new research is performed, reduce waste from unnecessary research lacking strong justification, and allow all stakeholders to remain up to date with collective knowledge and current understanding.

## Systematic online living evidence summaries (SOLES)

In addition to synthesis-ready research, evidence synthesis methodologies and outputs must be more efficient and accessible to empower researchers and other stakeholders to fully exploit these approaches. Evidence synthesis research and underlying infrastructures should be prepared to not only incorporate new research findings on a specific topic, but also to map research fields as their scope and focus evolve over time. Ideally, evidence synthesis should provide a continually up to date overview of a field, not simply a snapshot capturing a certain point in time.

Systematic Online Living Evidence Summaries (SOLES) are an emerging tool to harness AI technology and accelerate evidence synthesis. In SOLES, all existing evidence from a research domain is continuously gathered, synthesised and summarised to provide current curated content databases that can be interrogated via interactive web applications [63]. These platforms currently exist for fields including Alzheimer’s disease [64] and stroke [65] and provide comprehensive collections of preclinical animal studies automatically tagged with information such as the animal model and intervention administered, and quality criteria such as whether randomisation and blinding were carried out. SOLES give researchers an overview of evidence in a field, track study quality over time, and provide an accelerated starting point for more in-depth systematic reviews.

Working with researchers, funders, and others, evidence synthesists can improve how these evolving evidence summaries are curated and visualised for different stakeholders. For example, researchers may want to assess existing literature to refine modelling techniques or optimise outcome assessments for their experiments, while funders might want to identify evidence gaps to effectively direct resources or identify where certain questions have been sufficiently answered with high-quality evidence [63]. SOLES are the next step to allowing all decision-makers to access and synthesise data and in-depth experimental details at the click of a button to answer research questions.

## Community building

This integrated outlook speaks to the need for greater connectedness and collaboration between different stakeholders. Several groups are working to create the building blocks to integrate evidence synthesis into the research pipeline in preclinical biomedicine through (i) building researcher capacity for evidence synthesis through provision of training, (ii) making evidence synthesis research feasible and accessible with open infrastructure (e.g., [66]), and (iii) initiatives to coordinate efforts to address important challenges, e.g., the Collaborative Approach to Meta-Analysis and Review of Animal Data from Experimental Studies (CAMARADES) [67] and Communities for Open Research Synthesis (COReS) [68]. Institutional support in providing training and resources for evidence synthesis e.g., as core facilities, will be key [69, 70]. Working within and across communities has been critical to the success of open science initiatives [71], and we believe will be equally important in evidence synthesis to ensure solutions meet diverse needs [72–74].

## Challenges

Enacting a synthesis-enabled ecosystem is not without challenges. Some of the most pressing and difficult to address are existing research structures including individual incentives and rewards, and institutional cultures and climates. The research community lacks a widespread understanding of data as a collective resource and of sharing and synthesis as a benefit to all. These factors are active areas of research and explored by colleagues in-depth elsewhere [75].

Synthesis-ready data practices cannot be expected to be executed appropriately and integrated into routine practice without adequate support. We therefore call on funders and institutions to better support infrastructure and education for data management, protocol registration, and systematic review. Funders and institutions are uniquely placed, as they act as key levers for implementation by providing resources and supporting frameworks. A rigorous intervention is to mandate open science practices in grant applications, for example, Data Management and Sharing Plans (DMS Plans) are mandated according to the US National Institutes of Health (NIH) Data Management and Sharing Policy, effective from January 2023 [76]. Further, the introduction of practices to ensure compliance, such as withholding funding to researchers who have not yet deposited data or fail to meet open science standards, may be necessary, such as those outlined by the NIH Grants Policy Statement (Sect. 8.5 [77]).

Time will tell whether policies as pioneered by the NIH will drive significant improvement in compliance with FAIR data sharing. Lessons learned from the clinical trial space indicate that even when legal frameworks mandating sharing of trial data exist, compliance remains low [78]. This highlights the need to explore stronger or complementary strategies to ensure adherence. For example, the development and enforcement of EU-wide legal frameworks specifically addressing data sharing from animal studies could represent a substantial advancement in promoting open science (Table 1) [79]. This could include mandating open data for every publicly funded animal experiment with a timeframe for execution, for example, latest one year after the funding period has ended. Such a framework would likely be supported by animal welfare organisations. An example of stronger enforcement of mandates includes legal responsibility and repercussions for institutions and researchers not depositing and sharing all data gathered during research cycles. Other behaviour change strategies, such as creating incentives, fostering a culture of transparency, and providing ongoing training and resources, will be crucial to facilitating widespread and sustained adoption of policy and legislation [80].

Evidence synthesis itself is resource-intensive in terms of researcher time and the development of AI-driven approaches. Typically, funders invest heavily in resources to establish “islands” of information in preclinical research, but little investment is made to synthesise and integrate this evidence for decision-making. Encouragingly, there are signs the value of these methods is becoming more widely recognised. ZonMw, the Netherlands Organisation for Health Research and Development, and the German Federal Ministry of Education and Research (BMBF) both specifically fund preclinical systematic reviews. The Ensuring Value in Research (EViR) Funders’ Forum states that no new primary research should be conducted without appropriate overview of previous research (e.g., with a systematic review) [81].

AI, while holding great promise in the field of evidence synthesis, is a double-edged sword. Automation tools such as LLMs pose a threat to scientific integrity, with the falsification and especially fabrication of scientific data of immediate and growing concern. Retractions rose sharply in 2023, with sham papers and peer-review fraud cited among the reasons for the retraction of over 10,000 articles [82]. Advances in AI are feeding the generation of papers by paper mills, companies producing and selling fake research. AI delivers increased capacity to generate sham papers and increases the sophistication of falsified content, making it more difficult to detect. Publishers and journals are in a race to develop tools to tackle these problems. Meanwhile, the potential effect on conclusions if fraudulent data are included in preclinical evidence syntheses, our collective understanding of a research topic, is under investigation. Emerging research highlights this issue might substantially impact systematic review research [83], and may create a need for additional methodological approaches to ensure the provenance of included data [84].

## Concluding remarks

As evidence synthesists, we aim to connect education, community, and automation-facilitated infrastructure, to support a future vision where “*decision-makers are able to access up-to-date information instantaneously”*; they can easily access all information relevant to their research question, knowing that it is current because new research findings are synthesised as soon as they are created [85].

Synthesised preclinical evidence helps researchers most effectively plan the next steps in laboratory research programs, as well as informing steps through to clinical translation, research priority-setting exercises, and decision-making in healthcare guidelines and policy. Synthesis-ready practices need to be integrated into the design, conduct, and reporting of experimental research, and tools and infrastructure to support the conduct and dissemination of syntheses need to be established and optimised. These practices are inherently interconnected with open science and fostering an environment where research can be collaboratively scrutinised and built upon. Exploiting technology and automation techniques will be key to success, but changes to publication and funding models are also required to enable and facilitate these processes.

It is clear that an interdisciplinary and unified effort from multiple stakeholders is necessary to achieve an ecosystem where research outputs have the maximum impact on societal outcomes. Unified efforts will ensure that each stakeholder is supported by other links in the network and increase the chances of success for these improvement strategies.

## Data Availability

No datasets were generated or analysed during the current study.
